# fMRI-guided rTMS in the treatment of auditory verbal hallucinations in schizophrenia: a case report

**DOI:** 10.3389/fpsyt.2026.1752143

**Published:** 2026-01-30

**Authors:** Aykut Aytulun, Katia Brouzou, Mattia Campana, Navid Aliakbari, Francesca Pessanha-Schlegel, Timo Jendrik Faustmann, Milenko Kujovic, Daniel Kamp, Juha M. Lahnakoski, Leonhard Schilbach

**Affiliations:** 1 Department of Psychiatry and Psychotherapy, Medical Faculty, Heinrich-Heine-University Düsseldorf, Düsseldorf, Germany; 2 Department of General Psychiatry 2, LVR-Klinikum Düsseldorf, Düsseldorf, Germany; 3 Department of General Psychiatry 1, LVR-Klinikum Düsseldorf, Düsseldorf, Germany; 4 Institute of Neurosciences and Medicine, Brain and Behaviour (INM-7), Forschungszentrum Jülich, Jülich, Germany; 5 Institute of Systems Neuroscience, Medical Faculty, Heinrich-Heine-University Düsseldorf, Düsseldorf, Germany; 6 Department of Psychiatry and Psychotherapy, University Hospital, Ludwig Maximilians University Munich, Munich, Germany

**Keywords:** auditory verbal hallucinations, case report, fMRI, rTMS, schizophrenia

## Abstract

Auditory verbal hallucinations (AVH) are a core symptom of schizophrenia and contribute substantially to patient suffering and disability. They are among the most persistent symptoms and do not respond to medication in a substantial portion of patients. Here, we report the application of task-based functional magnetic resonance imaging (fMRI)-guided repetitive transcranial magnetic stimulation (rTMS) in a patient with treatment-resistant AVH. An individualized stimulation target was identified in the left temporal cortex near Heschl’s gyrus using a validated fMRI task. Over a period of 18 months, the patient underwent three cycles of inhibitory 1 Hz rTMS of this target. As a result, AVH severity decreased by 33% and the global symptom score improved by 40%. Functional connectivity analyses revealed an increase in coupling between the temporal target seed and a fronto-cingulate-insular network that has been implicated in reality and performance monitoring. This case highlights the potential of fMRI-guided rTMS as a personalized neuromodulatory therapy for refractory AVH in schizophrenia, warranting further systematic investigation.

## Introduction

Auditory verbal hallucinations (AVH), defined as hearing nonexistent spoken voices, are a typical symptom of schizophrenia (1). Unfortunately, 25-33% of patients with AVH do not respond to antipsychotic medication and 13% of patients suffer from debilitating daily acoustic hallucinations over a course of ten years (2). Ultra treatment resistant schizophrenia (UTRS), also termed clozapine-resistant schizophrenia, refers to patients who remain significantly symptomatic despite an adequate trial of clozapine, recent meta-analytic data show that UTRS is associated with persistent moderate to severe illness and a long disease duration (3, 4). The persistence of AVH can severely limit quality of life (5) and elevate the risk of self-harm or suicide (6).

Low frequency (≤1 Hz) repetitive transcranial magnetic stimulation (rTMS) of the left temporoparietal cortex has emerged as a promising therapeutic option, supported by a large multicenter randomized control trial (7) and meta analytic evidence (8, 9). Functional magnetic resonance imaging (fMRI) enables to precisely map brain regions involved in hallucinations like superior temporal gyrus and connecting networks. Recent studies have used resting state fMRI data to establish an imaging-navigated TMS treatment of AVH focused on the temporoparietal junction, which has proven to be effective (10). In spite of this progress, it still has to be determined which form of neuroimaging procedure is best suited to guide TMS while taking into account interindividual variability of heterogenous pathological networks (11). In this report, we present a novel approach which uses task-based fMRI to specifically focus on brain regions relevant for auditory processing to generate individualized TMS targets.

## Case presentation

CARE guidelines were followed (12) and informed consent for publication was obtained from the patient.

A 36-year-old Caucasian male patient with a 15-year history of schizophrenia as diagnosed in accordance with the International Classification of Diseases, 10^th^ Revision (F20.0) presented with persistent AVH despite antipsychotic medication consisting of 700 mg of clozapine and 1800 mg of valproic acid daily and long-acting injectable haloperidol decanoate 150 mg every 28 days. This antipsychotic medication was unchanged over the last 12 months and adequate adherence to treatment was ensured. Despite this, psychopathological symptoms including persecutory delusions, as well as thought insertion and thought broadcasting were observed. He had been admitted for more than twelve in-house treatments during the last three years due to clozapine-resistant AVH that consisted in imperative and commenting voices. The patient described these AVH as three distinct male voices, which were perceived as being from an external organization. The voices were derogatory and gave him commands to perform harmful actions. The patient, indeed, exhibited significant self-injurious behavior such as jumping from a bed which resulted in bilateral arm fractures. Considering clozapine-resistant AVH and in accordance with national guidelines, the patient had also received electroconvulsive therapy (ECT) over 16 sessions without sustained benefit.

In view of this situation, the patient was considered for rTMS in accordance with the German national guidelines for the treatment of schizophrenia (13). We decided to use fMRI-guided TMS in light of recent evidence that suggests that a neuroimaging-guided personalization of TMS targets can improve response rates (14, 15). Before treatment, the patient was assessed for TMS safety according to international guidelines (16). The timeline in **Table 1** summarizes the diagnostic and treatment procedures according to the CARE guidelines.

**Table 1:** Timeline of the three rTMS cycles - Brief Assessment of Cognition in Schizophrenia (BACS), Positive and Negative Symptome Scale (PANSS), Auditory Verbal Hallucinations Rating Scale (AVHRS), Clinical Global Impression-Severity (CGI-S) score.

Date	Event	Description	Findings	Psychopharmacological treatment
1. Month	1. Cycle of rTMS	Psychiatric assessment, fMRI-guided low-frequency rTMS (1 Hz, 1200 pulses/session) over left Heschl’s Gyrus, 8 sessions over 4 weeks (2/week)	Pre: PANSS (total score: 157), AVHRS (score: 49), BACS (z-score: -3.37), Post: subjective reduction of AVH, CGI-S at admission: 6, CGI-S at discharge: 4	Clozapine 700 mg/day; Valproic acid 1800 mg/day; long-acting injectable haloperidol decanoate 150 mg every 28 days
6. Month; Week 0	Baseline Evaluation	Psychiatric assessment Psychometry with PANSS, AVHRS, BACS fMRI for target localization (left temporoparietal cortex)	PANSS (total score: 131), AVHRS (score: 54), BACS (z-score: -3.01), CGI-S at admission: 5	Clozapine 700 mg/day; Valproic acid 1800 mg/day; long-acting injectable haloperidol decanoate 150 mg every 28 days
6./7. Month; Week 1–5	2. Cycle of rTMS	fMRI-guided low-frequency rTMS (1 Hz, 1200 pulses/session) over left Heschl’s Gyrus 9 sessions over 5 weeks (2/week)	Repeat fMRI (after session 4)	
7. Month; Week 6	Post-treatment evaluation	Psychometry with PANSS, AVHRS, BACS fMRI after 9. rTMS	PANSS (total score: 91), AVHRS (score:36), BACS (z-score: -2.94), CGI-S at discharge: 4	
12. Month	3. Cycle of rTMS	fMRI-guided low-frequency rTMS (1 Hz, 1200 pulses/session) over left Heschl’s Gyrus 9 sessions over 5 weeks (2/week)	Post: PANSS (total score:93), subjective reduction of AVH, CGI-S at admission: 6, CGI-S at discharge: 4	Clozapine 700 mg/day; Valproic acid 1800 mg/day; long-acting injectable haloperidol decanoate 150 mg every 28 days

**Figure 1:**
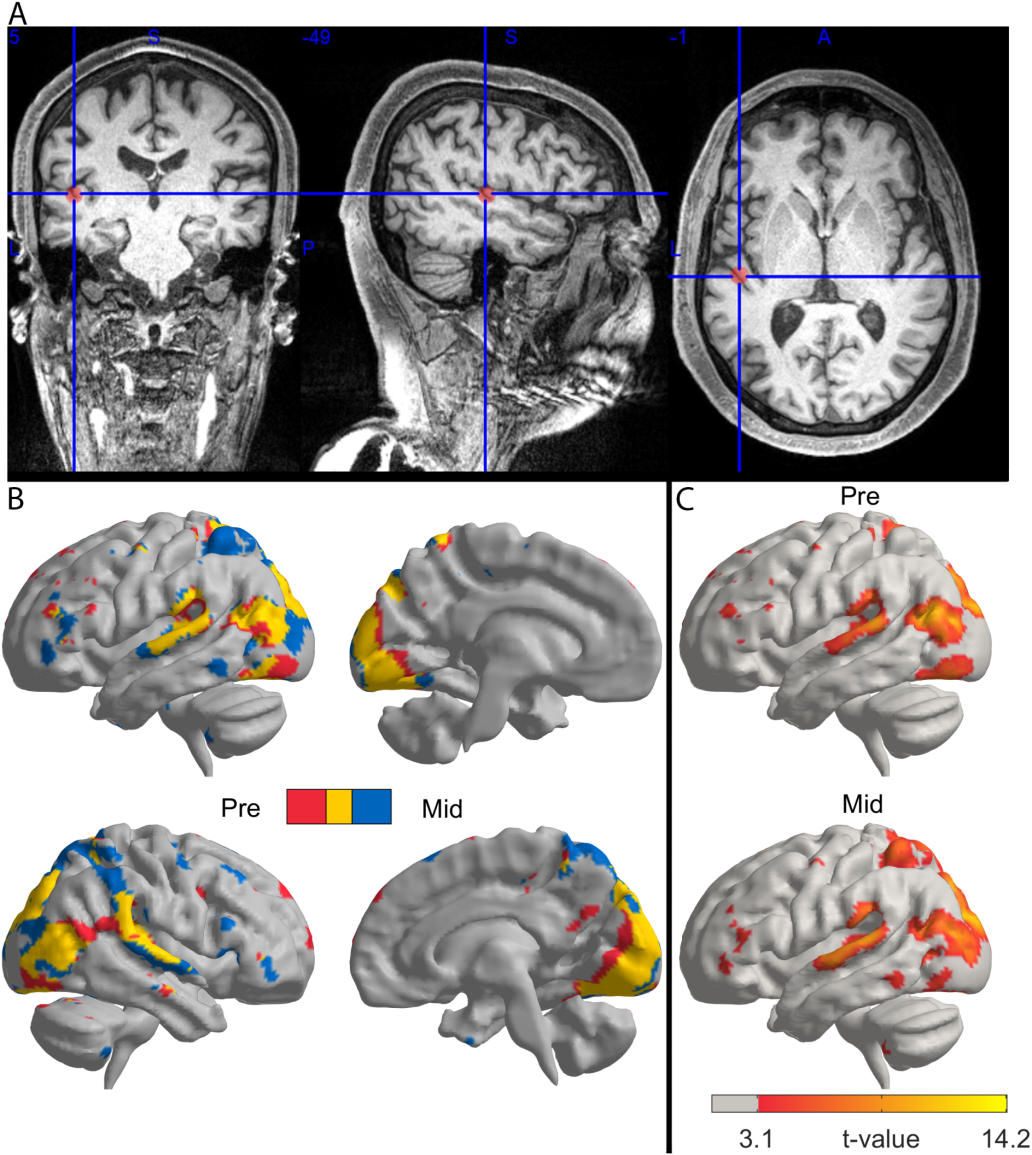
TMS target location & neural correlates of ‘social localizer’ task across treatment. **(A)** Coronal, sagittal, and axial slices of the patient’s T1-weighted MRI overlaid with the individualized fMRI peak. The crosshair marks the MNI coordinate of the stimulation target (x = –49, y = 5, z = -1), located in the left temporal cortex near Heschl’s gyrus. **(B)** Surface-rendered statistical maps of task-related brain activation related to sound loudness during the ‘social localizer’ paradigm before the first (Pre) and between the 4. and 5. (Mid) session of the second rTMS cycle. Overlay of pre (in red) and mid (in blue) treatment, yellow indicating an overlap of activations. **(C)** Statistical maps of the individual sessions before and after treatment (p< 0.001, uncorrected).

MRI measurements were obtained on a Siemens MAGNETOM Prisma 3.0 Tesla scanner using a 64-channel receiving coil including functional (EPI sequence, TR: 800 ms, TE: 37ms, Flip angle: 52°, Multiband acceleration factor: 8, matrix: 104 × 104, in-plane resolution: 2 mm x 2 mm. slice thickness: 2 mm, slice gap: 2 mm, slice count: 72, slice orientation: oblique) and T1 (MPRAGE sequence, TR: 2500ms, TE: 2.22ms, TI: 1000ms. Flip angle: 8°, matrix: 300 × 320, resolution: 0.8 mm isotropic, slice count: 208, slice orientation: sagittal) anatomical scans. Stimuli were controlled and synchronized with the scanner pulses with Presentation^®^ (Neurobehavioral Systems Inc., Berkeley, CA) software. The visual stimulation was delivered using a 40” MRI-compatible flatscreen display positioned inside the scanner room (InroomViewingDevice, NordicNeuroLab AS, Bergen, Norway) that could be seen via a mirror attached to the head coil. Auditory stimulations were delivered via Sensimetrics S14 insert earphones (Sensimetrics Corporation, Malden, Massachusetts, USA) driven by a t.amp S-100 MK II (Thomann GmbH, Burgebrach, Germany) analog power amplifier. Functional data were preprocessed and analyzed with SPM12 software (Statistical Parametric Mapping; https://www.fil.ion.ucl.ac.uk/spm). During preprocessing we removed the first four volumes, we realigned and unwarped the functional data and co-registered them to the structural image. Tissue segmentations were performed on the anatomical images. Finally, an 8 mm spatial smoothing full-width-at-half-maximum Gaussian kernel was applied to the functional images to reduce the effects of uncorrelated noise. Analyses were performed in the subject space to avoid biasing the activation loci by spatial normalization. Functional MRI (fMRI) was performed using a well-established ‘social localizer’ paradigm (17) to map regions related to auditory processing by calculating the root-mean-square (RMS) power of the audio track in windows equal to the TR of the fMRI sequence (800ms) and estimating the first-order effects of the model using a general linear model (GLM). This procedure allowed us to measure brain activation in the temporal lobe associated with variations in sound loudness (18), capturing responses to socially and linguistically complex auditory stimuli, such as voices with distinct identities and emotional content, which overlap with the phenomenology of AVH. The motion parameters estimated during preprocessing were included as nuisance parameters. The standard high-pass filter with a period of 128 seconds was applied to the model and the data in the analysis. The first-level results were thresholded at p<0.001 (uncorrected) at the voxel level. The peak activity within the left temporal cortex near Heschl’s Gyrus was chosen as the individual target (**Figure 1A**). We assessed positive, negative and general psychopathology symptoms with the Positive and Negative Syndrome Scale (PANSS) (19) and measured the severity and characteristics of AVH with the Auditory Vocal Hallucination Rating Scale (AVHRS) (20). Cognitive function was assessed using the Brief Assessment of Cognition in Schizophrenia (BACS) (21). Clinical status was assessed using the Clinical Global Impression-Severity (CGI-S) score (22).

Before the first cycle of rTMS, the patient had 49 points on the AVHRS score, a PANSS total score of 157 points, a Positive Symptom Score of 43 points, a negative symptom score of 39 points and a general psychopathology score of 75 points consistent with a severe clinical presentation of schizophrenia. The BACS assessment showed deficits on global cognitive performance (composite z-score = −3,37, T = 16).

Using Localite TMS navigator (Locality GmbH) the Power MAG Research 100 stimulator (MAG and More GmbH) with a PMG70 air cooled figure 8-coil, eight sessions of 1 Hz inhibitory rTMS (90% of resting motor threshold, 1200 pulses per session, 20 minutes per session) were applied to the left temporal cortex at the fMRI-derived stimulation target of Heschl’s gyrus over 4 weeks, resulting in two sessions per week and a total of 9600 pulses. The patient experienced a significant reduction of his AVH after three sessions and was discharged shortly after the rTMS cycle was completed. The CGI-S score improved from 6 = severely ill to 4 = moderately ill (**Table 1**). He was followed up on an outpatient basis for six months, maintaining an improved quality of life. After this period, he experienced a worsening of his AVH and was, therefore, readmitted for a second fMRI-guided rTMS cycle.

As part of the second cycle, nine sessions of 1 Hz inhibitory rTMS (90% of resting motor threshold, 1200 pulses per session, 20 minutes per session) were applied to the left temporal cortex at the fMRI target of Heschl’s gyrus over 5 weeks, resulting in two sessions per week and a total of 10800 pulses. fMRI was repeated mid-cycle (after session 4) and post-cycle (after session 9) to assess the effects of rTMS.

As before, the patient exhibited a clinically significant reduction in AVH in response to the second treatment. This was reflected in measurements on the AVHRS showing a reduction from 54 points at baseline before the second cycle to 36 points after rTMS (reduction of 33%). The PANSS total score decreased from 131 to 91, corresponding to a 40% reduction. Positive symptoms decreased by 40%, negative symptoms by 43%, and general psychopathology by 37%. The CGI-S score improved from 5 = markedly ill to 4 = moderately ill.

The BACS assessment showed no change in global cognitive performance (composite z-score = −2.94, T = 21), with slight improvements in speech domains regarding verbal fluency and verbal memory but a decline in motor speed and planning (**Table 2**).

**Table 2:** Brief Assessment of Cognition in Schizophrenia (BACS) during the second rTMS cycle.

BACS category	Pre-rTMS raw	Pre-rTMS z	Post-rTMS raw	Post-rTMS z
Verbal Memory	29	-2.06	37	-1.16
Digit Sequencing	21	-0.43	24	0.46
Token Motor Task	48	-2.50	40	-3.23
Verbal Fluency	26	-2.54	37	-1.53
Symbol Coding	27	-3.29	27	-3.29
Tower of London	19	0.44	14	-1.39
Composite Score	–	-3.01	–	-2.94

Data are presented in raw and z-scores pre and post rTMS. Z-Scores below -1 indicate impaired performance relative to norms.

Throughout the treatment, the patient was closely monitored for clinical changes and any side effects. The antipsychotic medication consisting of clozapine, valproic acid and long injectable haloperidol monthly was left unchanged during the entire duration of the treatment and was controlled via drug monitoring. The mean valproic acid level was 75 µg/mL (therapeutic range 50-100µg/ml) and the mean clozapine level was 575 ng/ml (therapeutic range 350–600 ng/ml). There were no relevant changes in the patient’s psychosocial environment, psychotherapy status, or the use of anxiolytic medication during the treatment cycles. The patient experienced activation of the masticatory muscles during rTMS, which was well tolerated. No adverse effects were reported, with only mild headaches occurring occasionally after the sessions, these side effects are among the most common side effects as noted in the rTMS safety guidelines (16).

After the second cycle of rTMS, the patient was again followed up on an outpatient basis for six months, maintaining a subjective good quality of life. After this period, he experienced a worsening of his AVH and was then readmitted for a third rTMS cycle. Here, nine sessions of 1 Hz inhibitory rTMS (90% of resting motor threshold, 1200 pulses per session, 20 minutes per session) were applied to the left temporal cortex at the fMRI target of Heschl’s gyrus over 5 weeks, resulting in two sessions per week and a total of 10800 pulses. The patient had a post-RTMS PANSS total score of 93 points, a Positive Symptom Score of 23 points, a negative symptom score of 24 points and a general psychopathology score of 46 points. The CGI-S score improved from 6 = severely ill to 4 = moderately ill. The patient’s reproducible improvement across all three rTMS interventions suggests a consistent treatment-response phenotype. Such stability of response across time and repeated stimulation supports the interpretation of a true neuromodulatory effect rather than nonspecific or placebo-related fluctuation.

### Neuroimaging analysis

In light of the consistent clinical response to TMS, we proceeded to investigate the underlying neural correlates by comparing the fMRI data obtained pre- and post-rTMS. To this end, we repeated the general linear model analysis in the MNI standard space with an otherwise identical procedure as for the TMS localization, except that the functional data were normalized to the standard space template. The resulting statistical maps for each condition (before first (Pre) and between the 4. and 5. (Mid) rTMS application) were rendered on a cortical surface with a threshold of p < 0.001 (uncorrected) (**Figures 1B, C**); post-rTMS results were similar but are omitted due to imprecise stimulus trigger timing, which caused the videos to start 16 seconds before the scanner, thus affecting the activation results.

This analysis demonstrated that pre-treatment activation during the ‘social localizer’ paradigm consisted of clusters of neural activity in auditory and visual regions, with peak activity centered around left temporal cortex including Heschl’s gyrus. Following rTMS, the overall task-related activation pattern across the brain, and in the left temporal cortex, remained stable (see **Figure 1**).

**Figure 2:**
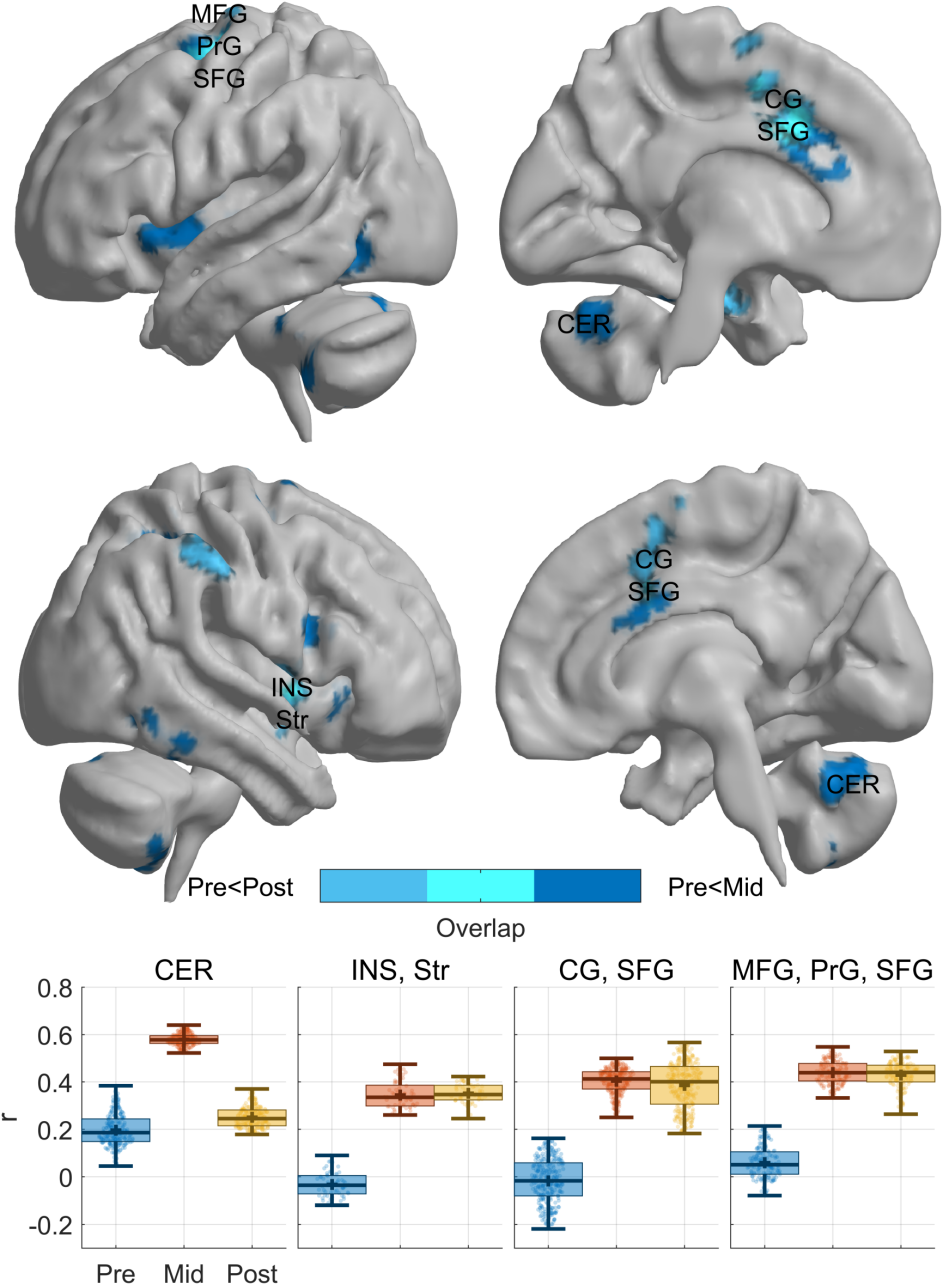
Functional connectivity change across treatment. Functional connectivity of the Heschl’s Gyrus Seed. Brain maps depict regions that exhibited significantly lower FC (p_847_<10^-6Bonferroni^) before (Pre), compared with between 4. and 5. rTMS session (Mid; dark blue) or after (Post; mid blue) treatment and their overlap (light blue). Functional connectivity values of the clusters showing differences in at least two pairwise comparisons (Pre-Mid, Pre-Post, Mid-Post) are depicted by boxplots below the brain maps. CER, Cerebellum; INS, Insula; Str, Striatum; CG, Cingulate Gyrus; SFG, Superior Frontal Gyrus; MFG, Middle Frontal Gyrus; PrG, Precentral Gyrus.

In addition to the activation-based analysis, we also performed a seed-based functional connectivity analysis for which the stimulation target in the left Heschl’s gyrus was used as the seed region. This analysis was done, because TMS effects are known to rely upon network-based effects that are induced via stimulation of one brain region (23). To do so, the mean BOLD time series was extracted from the seed, and voxel-wise Pearson correlations were calculated with all other voxels in the brain to identify regions showing coupled activity. We then tested for differences of functional connectivity between timepoints [parametric test of difference of Fisher’s Z-transformed correlation coefficients,p_847_<10^-6^,Bonferroni corrected; (24)]. This functional connectivity analysis demonstrated an enhanced connectivity with insula, superior temporal gyrus, cingulate gyrus, precentral gyrus and superior frontal gyrus during the mid- and post-cycle compared to the lack of connectivity pre-treatment (**Figure 2**). Additionally, we observed an increase in connectivity with the cerebellum during the mid-cycle fMRI.

## Discussion

This case report highlights the potential efficacy of fMRI-guided low frequency rTMS as a personalized treatment option for patients suffering from treatment-resistant AVH in schizophrenia. By using a novel, task-based fMRI approach to specifically focus on brain regions relevant for auditory processing, we were able to precisely target left temporal cortex using neuronavigated rTMS. This intervention reliably achieved a clinically meaningful reduction in auditory hallucinations and symptom severity after each of three rTMS cycles in our patient, who had not responded to a combined medication of clozapine, valproic acid and haloperidol.

This finding stands in contrast to a meta-analysis pooling 131 clozapine-treated patients across ten randomized controlled trials which did not find any improvement after rTMS for auditory hallucinations or negative symptoms (25). However, the optimal rTMS protocol to treat AVH remains unclear, although continuous theta burst stimulation has been shown to be non-inferior to conventional 1 Hz stimulation (26). A large multi-center, randomized, sham-controlled, triple blind phase 3 trial has recently shown that sequential bilateral temporo-parietal TBS without fMRI guidance over three weeks was safe and effective for reducing AVH in adults with schizophrenia (7).

Also, the literature on neuroimaging-guided TMS for AVH – in contrast to an increasing number of studies for fMRI-guided TMS in depression - remains limited. To our knowledge, there are only very few studies that have used fMRI-guided rTMS in AVH (11). Moreover, the heterogeneity of the neuroimaging data and the limited number of studies has not allowed to distinguish its effect in comparison to non-personalized targeting methods. One recent trial demonstrated benefits of fMRI-guided targeting of the left temporoparietal junction (10). Our case employs task-based fMRI to functionally localize primary auditory regions as those have been implicated in ‘symptom capture’ neuroimaging studies (27–30).

Despite the marked improvement in AVHRS and PANSS scores due to rTMS, cognitive performance (BACS) remained largely unchanged, with minor declines in executive function. This dissociation suggests that rTMS on the auditory cortex involves partially independent neural circuits. While inhibitory 1 Hz stimulation over the auditory cortex could theoretically affect fronto-temporal networks, fatigue or daily illness-related fluctuation could contribute to the decline in the subtests.

In addition to these auditory seed regions, we also investigated functional connectivity patterns based on the idea that TMS effects typically rely on changes in entire neural networks that are brought about by stimulating one hub of the network (31, 32). Interestingly, our findings bear resemblance to previous studies, which have shown that rTMS to left temporoparietal junction reduced activation not only locally but also in bilateral auditory cortices, insula, and anterior cingulate (33). Another structural connectivity study described, similar to our results, connections between Heschl’s Gyrus and the insular cortex, cingulate gyrus and the cerebellum to be relevant (34). The increased functional connectivity between the Heschl’s gyrus and the cerebellum during the mid-cycle may reflect either a compensatory mechanism, helping to suppress internally generated auditory signals (35), or a partial restoration of sensory filtering impaired in schizophrenia (36). Given the single-case nature of this report, it is not possible to determine which mechanism predominates, which should be addressed in future studies. A more recent study has shown that prior to low-frequency rTMS of the left temporoparietal junction, patients displayed altered functional connectivity of the superior frontal gyrus, which was reversed after rTMS (37). Importantly, these findings appear to tie in with recent results from Djikstra et al. (38), who conducted a study to investigate the neural basis of distinguishing imagination from reality, arguably a process relevant for understanding AVH: they found that a similar network as the one implicated in our case is relevant for reality-monitoring, decision confidence and ambiguity assessments. Although they did not use brain stimulation, these results map onto the exact same network that TMS appears to influence when used for the treatment of hallucinations.

Clearly, this case report has limitations: First, the findings are based on a single patient and comparisons of functional connectivity rely on single parametric tests of single timepoints, which limits generalizability. Second, there was no sham control, making it difficult to rule out placebo effects. No data on longer term effects is available so far. Third, we acknowledge that the post-treatment functional connectivity analysis is confounded by a temporal mismatch, so that the task-state connectivity may not perfectly reflect the intended experimental condition due to the acquisition lag. On the other hand, the durability of the TMS effects is highlighted by the patient’s ability to sustain an improved quality of life as an outpatient for around six months in between each rTMS cycle for a total of 18 months so far. In light of these findings, we argue that randomized controlled trials are desperately needed to compare fMRI-guided stimulation protocols to standard TMS protocols without fMRI guidance in order to determine whether targeting based on functional neuroimaging offers clinically relevant advantages in schizophrenia, similar to how this is being evaluated in the case of depression (23, 39).

### Patient’s perspective

“Following the stimulation, I have noticed a clear improvement after three sessions. The three voices have become less aggressive towards me, and their loudness has decreased by approximately 30%. They no longer make hurtful comments during the day and no longer cause sensory overload, allowing me to think more clearly. I am now able to engage more easily in daily activities, such as shopping or other routine tasks. The voices no longer speak about me in the third person but address me directly, and they take turns rather than speaking over one another. Overall, I am thankful that this procedure helps me, and I feel that I have gained greater control over my own life”.

## Data Availability

The original contributions presented in the study are included in the article/Supplementary Material. Further inquiries can be directed to the corresponding authors.
